# Assessment of primary open-angle glaucoma peripapillary and macular choroidal area using enhanced depth imaging optical coherence tomography

**DOI:** 10.1371/journal.pone.0231214

**Published:** 2020-04-06

**Authors:** Hirokazu Kojima, Kazuyuki Hirooka, Eri Nitta, Shozo Sonoda, Taiji Sakamoto, Yoshiaki Kiuchi

**Affiliations:** 1 Department of Ophthalmology, Kagawa University Faculty of Medicine, Kagawa, Japan; 2 Department of Ophthalmology and Visual Science, Graduate School of Biomedical Sciences, Hiroshima University, Hiroshima, Japan; 3 Department of Ophthalmology, Kagoshima University Graduate School of Medical and Dental Sciences, Kagoshima, Japan; Massachusetts Eye & Ear Infirmary, Harvard Medical School, UNITED STATES

## Abstract

**Purpose:**

The current study investigated differences in the peripapillary and macular choroidal areas between patients with primary open-angle glaucoma (POAG) and healthy controls because the choroid may potentially play a role in glaucoma pathophysiology.

**Methods:**

We assessed 57 healthy controls and 42 POAG patients in a cross-sectional comparative study. We used enhanced depth imaging optical coherence tomography (EDI-OCT) and then converted the luminal and interstitial areas to binary images using the Niblack method to obtain peripapillary and macular choroidal images. The relationship between the choroidal area and demographic and ocular characteristics were determined with univariate and multivariate linear regression analysis.

**Results:**

Regarding the peripapillary choroidal area, no significant differences were noted between healthy controls and POAG patients (1,836,336 ± 605,617 μm^2^ vs. 1,775,566 ± 477,317 μm^2^, respectively, *P* = 0.60). There were also no differences found for the macular choroidal area (controls: 347,220 ± 115,409 μm^2^, patients: 342,193 ± 104,356 μm^2^, *P* = 0.83). Multivariate regression analysis in the POAG patients revealed there was a significant relationship between the macular choroidal area and age (β = −0.525, *P* = 0.002) and axial length (β = −0.458, *P* = 0.005). In contrast, no correlation was found between peripapillary choroidal areas and various attributes in the POAG patients.

**Conclusions:**

EDI-OCT showed no differences in the peripapillary or macular choroidal area in healthy individuals compared to POAG patients.

## Introduction

The most abundant blood vessel layer in the eye is the choroid, which has the most abundant blood flow in the body per unit tissue weight.[1] The microvasculature of the peripapillary choroid is an important consideration in glaucoma because short posterior ciliary arteries supply the choroid and the optic nerve head. Thus, understanding the pathophysiological mechanisms of glaucoma requires assessment of the choroid.

New techniques have been introduced, including enhanced depth imaging (EDI) spectral domain optical coherence tomography (OCT), which allow cross-sectional imaging of the choroid.[2] Several studies have used these methods and described relationships between the thickness of the choroid and glaucoma. Some reports have shown no differences in choroidal thickness in healthy compared to glaucoma patients,[3–6] whereas others have shown a thinner choroid in glaucoma patients.[7–9] These previous studies measured the thickness of the choroid in a small area (at 1.7 mm superior, temporal, inferior, and nasal to the optic disc center and at 1- and 3-mm nasal, temporal, superior, and inferior to the fovea). In our previous studies, we assessed a 1,500-μm wide macular choroid and a 1.7-mm area of the peripapillary choroid near the middle of the optic nerve disc[10–12], thus allowing us to obtain more information about the choroid.

Intraocular pressure (IOP) is considered the main risk factor for primary open-angle glaucoma (POAG). However, in normal-tension glaucoma (NTG), increased IOP is less important.[13] Thus, factors other than the IOP should also be considered when evaluating glaucoma. As the choroid has a significant role with regard to the ocular blood flow, it is thought to be important in the pathophysiology of glaucoma.[14] Our previous study showed a smaller peripapillary choroidal area in patients with NTG.[11] Here we compared the area of the peripapillary and macular choroid in healthy vs. POAG eyes.

## Materials and methods

### Patients

This cross-sectional comparative study evaluated participants who underwent testing at Kagawa University Hospital between July 2018 and March 2019. The study was conducted according to the principles of the Declaration of Helsinki. Each patient was provided a detailed explanation of the study and gave written informed consent. The Kagawa University Faculty of Medicine Institutional Review Board approved the study’s protocol.

All patients completed a full ophthalmic examination in which the central corneal thickness (CCT), central and peripheral fields, gonioscopy, slit lamp, and visual acuity were assessed with refraction. Age- and axial length-matched healthy control volunteers underwent the same measurements. Patients were enrolled only if they had a spherical error between +4.0 and −6.0 diopters (D) and a cylinder within ±2.0 D. Eyes with glaucoma were considered those with glaucomatous optic disc changes (thinning of the neuroretinal rim, notching, or a defect in the retinal nerve fiber layer) and visual field loss. Glaucomatous visual field loss was defined as at least two consecutive baseline hemifield tests were outside the normal limits. This definition included at least three contiguous test points in the same hemifield with a pattern deviation plot at *P* < 1%, and at least one test point at *P* < 0.5%. A POAG diagnosis was defined as an untreated IOP of >21 mmHg. We used the same inclusion and exclusion criteria reported in a previous study.[11] One inclusion criterion was open angles. Patients with a history of retinal diseases, poor image quality due to unstable fixation, severe cataracts, or a history of previous laser therapy were excluded. The same researcher performed all EDI-OCT examinations. Only one eye, typically the right eye, was examined in each patient to exclude individual variations. If the right eye did not meet the inclusion criteria, we examined the left eye.

### EDI-OCT

The Heidelberg Spectralis (Heidelberg Engineering, Heidelberg, Germany) with the EDI-OCT technique was used to capture the macular or peripapillary choroidal images.[10–12] All measurements were obtained between 13:00 and 15:00 hours. All eyes were examined without mydriasis. The macular region images were captured using seven horizontal lines of 30 × 10° through the center of the fovea. The peripapillary region images were obtained using a 360° circle that was 3.4 mm in diameter that was centered on the optic disc. The best quality image from at least three scans was used for assessment. Choroidal thickness was considered to be the area between the outer portion of the hyperreflective line that corresponded to the retinal pigment epithelium and the inner surface of the sclera. The manual segmentation function was used because Spectralis OCT does not provide an algorithm for automatic segmentation of the choroid.

### Binarization of the choroid EDI-OCT images

**Fig 1:**
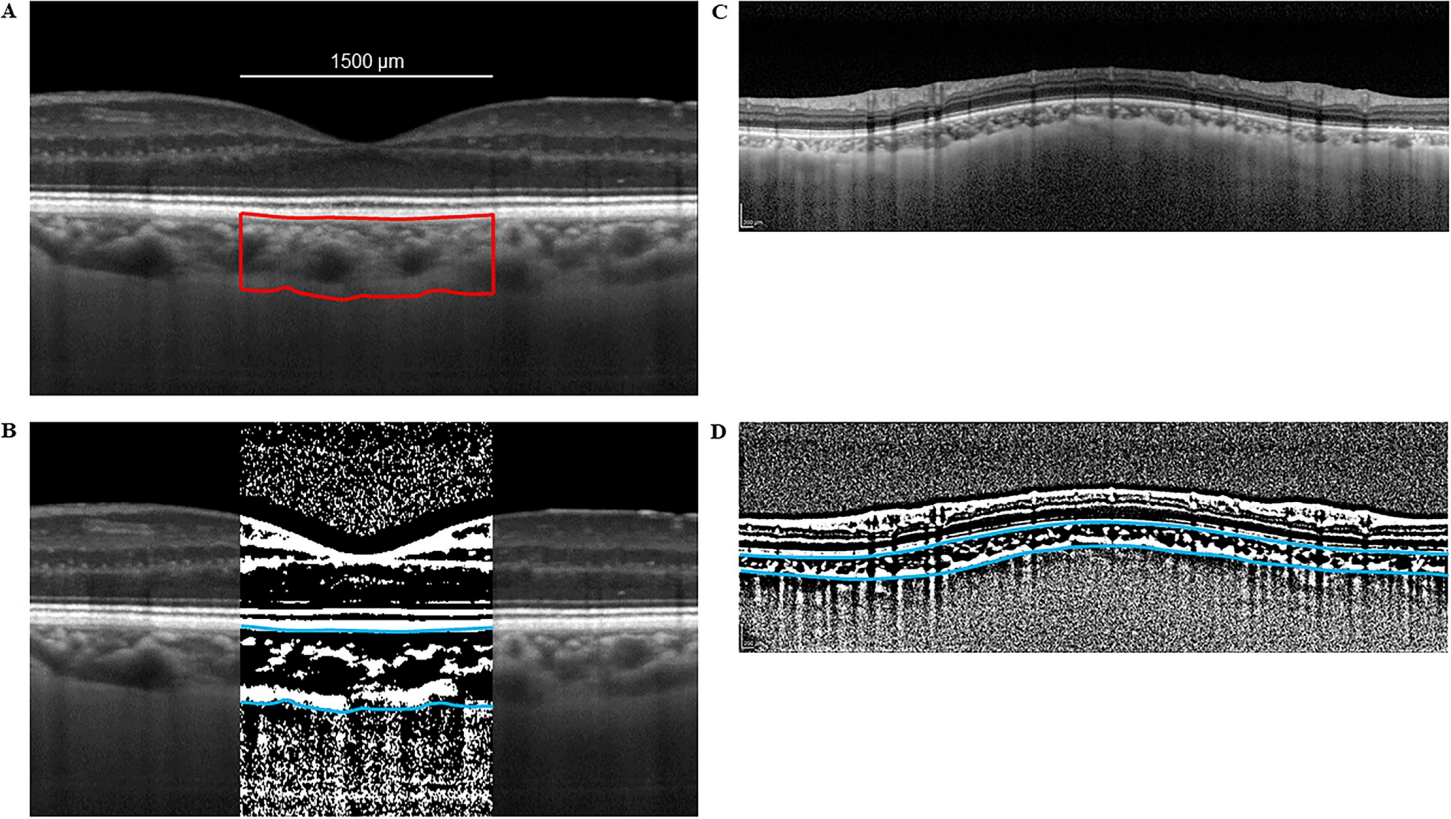
Enhanced depth imaging OCT image and converted binary image of the eye of a glaucoma patient. The EDI-OCT images in the macular area (A) or the peripapillary area (C) were converted to binary images (B, D) using ImageJ software. The luminal (dark area) and interstitial areas are seen. The area between the blue lines indicates the measurement area of the choroid.

The best EDI-OCT images were recorded, masked, and displayed on a computer screen. One author (HK) examined each image. The choroidal area in each EDI-OCT image was binarized using the modified Niblack method.[15] Briefly, the area of the macular choroid that was 1,500 μm wide and that extended vertically (Fig 1A and 1B) in EDI-OCT images was first assessed using ImageJ software (version 1.47, NIH, Bethesda, MD).[10–12] The examined region included a 1.7-mm area around the optic nerve disc center (Fig 1C and 1D) that ranged from the retinal pigment epithelium to the chorioscleral border.[10–12] Areas were delineated with ImageJ ROI Manager. We averaged the reflectivities of the lumens of three randomly selected choroidal vessels with lumens >100 μm that were selected using the Oval Selection Tool on the ImageJ tool bar. To reduce the noise in the OCT image, this average reflectivity was defined as the minimum value. The image was converted, adjusted to 8 bits using the Niblack Auto Local Threshold program, and then the binarized image was converted to a RGB image because of technical requirements for binarization and the automated calculations performed by ImageJ. The Threshold Tool was used to identify the hyporeflective area, with dark and light pixels considered hyporeflective and hyperreflective areas, respectively. Information was added about the relationship between the distance on the fundus and the pitch of the pixels in the EDI-OCT images, which depend on the axial length, and then the hyperreflective and hyporeflective areas were automatically calculated. Light and dark pixels were considered the interstitial choroid and the luminal area, respectively.

### Statistical analysis

The sample size was chosen according to our previous report[10] and was calculated by assuming a 10% variation in the peripapillary choroidal area between the POAG and healthy group and a 1:1 ratio of glaucomatous to normal eyes. The sample size needed was 33 per group for a predictive power of 90%.

JMP software version 14 (SAS Inc., Cary, NC) was used for statistical analyses. The independent Student’s *t*-test was performed to assess differences in age, IOP, systolic blood pressure (SBP), diastolic blood pressure (DBP), ocular perfusion pressure (OPP), CCT, and axial length. Differences in the categorical parameters were assessed using the χ^2^ test. OPP was calculated as OPP = 2/3[DBP + 1/3(SBP–DBP)]–IOP. Univariate and multivariate linear regression was used to assess the relationship between the choroidal area and different parameters including age, axial length, CCT, IOP, DBP, and OPP in the POAG and healthy groups. Variables with *P* < 0.2 in the univariate regression were included in the multivariate regression. *P* < 0.05 was considered statistically significant. All data are shown as the mean ± standard deviation (SD).

## Results

Clinical characteristics of the enrolled subjects are presented in Table 1. The mean age of the healthy controls and POAG patients was 64.9 ± 11.9 years (range, 40–85 years) and 67.6 ± 10.1 years (range, 34–86 years), respectively (*P* = 0.36). No significant differences were observed for gender (*P* = 0.16), IOP (*P* = 0.06), axial length (*P* = 0.07), or OPP (*P* = 0.85).

**Table 1:** Clinical characteristics of healthy controls and primary open-angle glaucoma patients.

	Healthy	POAG	*P* value
Age (y)	64.9 ± 11.9	67.6 ± 10.1	0.36
Gender (M/F)	23/34	23/19	0.16
IOP (mmHg)	14.9 ± 3.3	16.1 ± 2.9	0.06
Axial length (mm)	24.2 ± 1.7	24.8 ± 1.8	0.07
Central corneal thickness (μm)	526.2 ± 38.7	528.9 ± 53.9	0.78
Ocular perfusion pressure (mmHg)	47.3 ± 7.2	47.6 ± 7.6	0.85
Initial systemic blood pressure (mmHg)			
Systolic	127.8 ± 15.7	129.4 ± 16.8	0.63
Diastolic	76.0 ± 11.7	78.6 ± 11.1	0.27
Diabetic mellitus (%)	9 (15.8)	8 (19.0)	0.67
Hypertension (%)	27 (47.4)	16 (38.1)	0.36
Glaucoma history			
Mean deviation (dB)		-15.41 ± 7.41	
No. of glaucoma medications		3.3 ± 0.9	
IOP at first visit (mmHg)		18.6 ± 4.9	

POAG; primary open-angle glaucoma, M; male, F; female, IOP; intraocular pressure

Mean peripapillary choroidal areas were 1,836,336 ± 605,617 μm^2^ and 1,775,566 ± 477,317 μm^2^ in the healthy controls and POAG patients, respectively (*P* = 0.60) (Table 2). Peripapillary luminal and interstitial areas were 1,029,898 ± 430,908 μm^2^ and 806,437 ± 183,512 μm^2^ in the healthy controls and 994,971 ± 330,883 μm^2^ (*P* = 0.67) and 780,595 ± 164,300 μm^2^ (*P* = 0.48) in POAG patients, respectively (Table 2). Macular choroidal, luminal, and interstitial areas were 347,220 ± 115,409 μm^2^, 226,191 ± 81,621 μm^2^, and 121,029 ± 36,899 μm^2^ in the healthy controls and 342,193 ± 104,356 μm^2^, 225,314 ± 72,722 μm^2^, and 116,878 ± 35,532 in POAG patients, respectively (Table 2). We found no significant differences between the healthy controls and POAG patients.

**Table 2:** Choroidal area observed in EDI-OCT images.

	Peripapillary choroidal area			Macular choroidal area		
	Healthy	POAG	*P* value	Healthy	POAG	*P* value
Total area (μm^2^)	1836336 ± 605617	1775566 ± 477317	0.60	347220 ± 115409	342193 ± 104356	0.83
Luminal area (μm^2^)	1029898 ± 430908	994971 ± 330883	0.67	226191 ± 81621	225314 ± 72722	0.96
Interstitial area (μm^2^)	806437 ± 183512	780595 ± 164300	0.48	121029 ± 36899	116878 ± 35532	0.58

POAG; primary open-angle glaucoma

Subsequently, we then divided the POAG patients into two groups, the uncontrolled/progressive and the controlled group. Mean peripapillary choroidal areas were 1,731,441 ± 424,960 μm^2^ (*P* = 0.63, Dunnett t-test, as compared to healthy controls) in the uncontrolled/progressive POAG patients (n = 32) and 1,916,767 ± 641,953 μm^2^ (*P* = 0.89) in the controlled POAG patients (n = 10). Macular choroidal areas were 327,789 ± 94,926 μm^2^ (*P* = 0.67) in the uncontrolled/progressive POAG patients and 388,282 ± 129,172 μm^2^ (*P* = 0.48) in the controlled POAG patients.

Univariate regression revealed a significant negative relationship with age for the peripapillary choroidal area of the healthy controls (β = −0.483, *P* = 0.0002). Univariate regression additionally indicated a significant association with age (Healthy: β = −0.272, *P* = 0.04, POAG: β = −0.312, *P* = 0.04) for the macular choroidal area of the healthy controls and POAG patents (Table 3). Multivariate regression analysis demonstrated a significant relationship for age (β = −0.419, *P* = 0.002), axial length (β = −0.512, *P* = 0.0002), and CCT (β = 0.303, *P* = 0.01) with the macular choroidal area in healthy controls (Table 4). In addition, we identified a significant relationship between the macular choroidal area and age (β = −0.525, *P* = 0.002) and axial length (β = −0.458, *P* = 0.005) in the POAG patients. Univariate regression revealed a significant negative relationship with age for the peripapillary luminal or interstitial area of the healthy controls (luminal: β = −0.460, *P* = 0.0004, interstitial: β = −0.515, *P* < 0.0001, Tables 5 and 6). Multivariate regression analysis indicated that there was a significant relationship for age and axial length with the macular choroidal luminal area in the healthy controls and POAG patients (Table 7). Furthermore, multivariate regression analysis also demonstrated that there was a significant relationship for age, axial length and CCT with the macular choroidal interstitial area in healthy controls and for age with the macular choroidal interstitial area in the POAG patients (Table 8).

**Table 3:** Univariate regression analysis of choroidal area with associated factors.

	Peripapillary choroidal area				Macular choroidal area			
	Healthy		POAG		Healthy		POAG	
	β	*P* value	Β	*P* value	Β	*P* value	β	*P* value
Age	-0.483	0.0002	-0.258	0.10	-0.272	0.04	-0.312	0.04
Axial length	-0.015	0.91	-0.129	0.41	-0.269	0.04	-0.215	0.17
CCT	-0.047	0.73	-0.029	0.86	0.199	0.14	-0.059	0.72
IOP	0.088	0.52	0.06	0.71	0.073	0.59	-0.046	0.77
OPP	-0.023	0.87	0.247	0.43	0.061	0.65	0.142	0.37
BP								
Systolic	-0.058	0.67	0.169	0.28	0.154	0.25	0.192	0.22
Diastolic	0.064	0.64	0.101	0.53	0.028	0.84	0.047	0.77

POAG; Primary open-angle glaucoma, CCT; Central corneal thickness, IOP; Intraocular pressure, OPP; Ocular perfusion pressure

BP; Blood pressure

**Table 4:** Multivariate regression analysis of macular choroidal area with associated factors.

	Healthy			POAG		
	β	*P* value	VIF	β	*P* value	VIF
Age	-0.419	0.002	1.185	-0.525	0.002	1.274
Axial length	-0.512	0.0002	1.256	-0.458	0.005	1.274
CCT	0.303	0.01	1.066			

POAG; Primary open-angle glaucoma, CCT; Central corneal thickness, VIF; Variance inflation factor

**Table 5:** Univariate regression analysis of choroidal luminal area with associated factors.

	Peripapillary luminal area				Macular luminal area			
	Healthy		POAG		Healthy		POAG	
	β	*P* value	β	*P* value	β	*P* value	β	*P* value
Age	-0.460	0.0004	-0.275	0.08	-0.276	0.04	-0.291	0.06
Axial length	-0.052	0.70	-0.168	0.29	-0.301	0.02	-0.254	0.10
CCT	-0.037	0.79	-0.066	0.68	0.153	0.26	-0.061	0.70
IOP	0.105	0.44	0.059	0.71	0.058	0.67	-0.015	0.92
OPP	-0.027	0.85	0.137	0.39	0.069	0.61	0.121	0.45
BP								
Systolic	-0.043	0.75	0.182	0.25	0.160	0.24	0.167	0.29
Diastolic	0.059	0.67	0.110	0.49	0.026	0.85	0.052	0.74

POAG; Primary open-angle glaucoma, CCT; Central corneal thickness, IOP; Intraocular pressure, OPP; Ocular perfusion pressure

BP; Blood pressure

**Table 6:** Univariate regression analysis of choroidal interstitial area with associated factors.

	Peripapillary interstitial area				Macular interstitial area			
	Healthy		POAG		Healthy		POAG	
	β	*P* value	β	*P* value	β	*P* value	β	*P* value
Age	-0.515	<0.0001	-0.196	0.21	-0.241	0.07	-0.322	0.04
Axial length	0.072	0.60	-0.037	0.82	-0.176	0.19	-0.111	0.48
CCT	-0.070	0.61	0.049	0.76	0.287	0.03	-0.047	0.77
IOP	0.045	0.74	0.056	0.73	0.101	0.45	-0.103	0.52
OPP	-0.012	0.93	0.086	0.59	0.036	0.79	0.169	0.28
BP								
Systolic	-0.090	0.51	0.125	0.43	0.127	0.35	0.222	0.16
Diastolic	0.072	0.60	0.072	0.65	0.030	0.82	0.033	0.84

POAG; Primary open-angle glaucoma, CCT; Central corneal thickness, IOP; Intraocular pressure, OPP; Ocular perfusion pressure

BP; Blood pressure

**Table 7:** Multivariate regression analysis of macular choroidal luminal area with associated factors.

	Healthy			POAG		
	β	*P* value	VIF	β	*P* value	VIF
Age	-0.580	<0.0001	1.206	-0.520	0.002	1.274
Axial length	-0.292	0.03	1.206	-0.495	0.003	1.274

POAG; Primary open-angle glaucoma, VIF; Variance inflation factor

**Table 8:** Multivariate regression analysis of macular choroidal interstitial area with associated factors.

	Healthy			POAG		
	β	*P* value	VIF	β	*P* value	VIF
Age	-0.334	0.01	1.185	-0.305	0.047	1.008
Axial length	-0.34	0.005	1.256			
CCT	0.367	0.005	1.066			
SBP				0.196	0.20	1.008

POAG; Primary open-angle glaucoma, CCT; Central corneal thickness, SBP; Systolic blood pressure, VIF; Variance inflation factor

## Discussion

Here we assessed the peripapillary and macular choroidal area in POAG patients compared to healthy controls. In NTG patients, we recently reported decreases in the peripapillary choroidal area,[11] suggesting that the peripapillary choroidal area is crucial to the pathogenesis of NTG.

NTG and POAG fall on a continuum of open-angle glaucoma. The main difference is that a sufficiently high IOP is the most important risk factor in POAG, and other factors in addition to IOP play an important role in NTG. Therefore, understanding the difference in pathology between NTG and POAG is important. NTG and POAG should be considered separately in studies of the pathology of glaucoma. Most OCT studies found no difference in the thickness of the peripapillary choroid between glaucoma patients and healthy controls.[3,4,16–19] However, three studies[3,17,18] examined high-tension glaucoma patients, and one study[18] examined NTG patients. One study[4] included patients with both POAG and primary angle closure glaucoma (PACG), and another[16] did not report the glaucoma type. Some OCT studies showed a significantly thinner peripapillary choroid in glaucoma compared to healthy eyes.[7–9] Two studies[7,8] examined patients with NTG, and one[9] examined 114 NTG patients and 20 POAG patients. Thus, the study assessing NTG patients showed a significantly thinner peripapillary choroid in these patients. We recently found a significant difference in the peripapillary choroidal area between healthy controls and NTG patients, even though the macular choroidal area was similar in these groups.[11] In our present study, we did not find a difference in the peripapillary choroidal area in healthy controls compared to POAG patients. When we analyzed a direct POAG vs NTG, there was no significant difference in peripapillary choroidal are (1,775,566 ± 477,317 μm^2^ vs 1,606,448 ± 418,214 μm^2^, *P* = 0.11). Probably number of NTG patients were not enough (n = 35). The choroid supplies nearly three quarters of the eye’s circulating blood as well as nutrients to the outer retina and optic nerve head, especially the prelaminar region, which is involved in death of retinal ganglion cells in patients with glaucoma.[20] The peripapillary choroidal area may play a more important role in glaucoma than the macular choroidal area because the former supplies blood to the optic nerve. Thus, the crucial difference in the pathology between POAG and NTG may be associated with the optic nerve blood supply.

We did not find an association between age and the peripapillary choroidal area in POAG patients, but a negative correlation was found in healthy controls. There was a significantly thinner peripapillary choroidal thickness in patients with glaucoma who had sclerotic optic disc damage as compared to that observed in patients with focal or diffuse optic disc damage or the healthy controls.[21] We assume that the presence of sclerotic optic disc damage in the POAG patients was related to the lack of an association observed between the age and the peripapillary choroidal area.

Macular choroidal area, except macular choroidal interstitial area, was correlated with age and axial length in POAG patients and healthy controls, which agrees with findings in most previous choroidal thickness studies.[4–6,9,22,23] Therefore, it is essential that both age and axial length are taken into account when choroidal area (thickness) is evaluated.

Our current study has some limitations. First, we had to identify Bruch’s membrane and the inner scleral border manually, because no OCT software can perform automated segmentation. Second, all glaucoma patients had a history of receiving anti-glaucoma drugs, which may impact choroidal thickness. Anti-glaucoma drugs can increase the subfoveal choroidal thickness in POAG patients.[24] However, here we only measured choroidal thickness in the subfoveal region and the parafoveal area; we did not assess peripapillary choroidal thickness, which may be associated with glaucoma.

In conclusion, healthy and POAG patients have similar peripapillary and macular choroidal areas as determined with EDI-OCT.

## Supporting information

S1 Data (XLSX)
